# A Five-Locus SSR Molecular-Affinity Framework Provides Redundancy Context for Previously Identified Elite-Relevant Lines in a ‘Morita II’-Derived *Stevia rebaudiana* Breeding Collection

**DOI:** 10.3390/ijms27125277

**Published:** 2026-06-10

**Authors:** Luis Alfonso Rodríguez-Páez, Yirlis Yadeth Pineda-Rodriguez, Edna Judith Marquez-Fernandez, Alfredo Jarma-Orozco

**Affiliations:** 1 Laboratorio de Biología Molecular Aplicada, Facultad de Ciencias Agrícolas, Universidad de Córdoba, Montería 230002, Colombia; yirlispinedar@correo.unicordoba.edu.co (Y.Y.P.-R.); ajarma@correo.unicordoba.edu.co (A.J.-O.); 2 Laboratorio de Biología Molecular y Celular, Facultad de Ciencias, Universidad Nacional de Colombia, Sede Medellín, Medellín 050034, Colombia; ejmarque@unal.edu.co

**Keywords:** clonal breeding, molecular affinity, germplasm redundancy, line retention, redundancy screening, SSR-based discrimination, *Stevia rebaudiana*, tropical crop improvement

## Abstract

The molecular management of elite-relevant lines in clonally exploited crops requires more than broad genetic structure alone. In *Stevia rebaudiana*, breeding materials derived from cv. ‘Morita II’ may retain useful variation while also concentrating molecularly similar lines, increasing redundancy within selection pipelines. This study assessed whether a reduced five-locus SSR dataset could provide an operational molecular-affinity framework for redundancy screening and breeding-context interpretation of previously identified elite-relevant lines in a ‘Morita II’-derived breeding collection. A curated five-locus SSR dataset comprising 85 genotypes from a tropical breeding programme was analysed using the Wang relatedness estimator, operational molecular-affinity classes, UPGMA clustering based on Wang-derived dissimilarity and permutation-based assessment of mean Wang relatedness. The collection combined a broad fraction of comparisons showing no detectable positive molecular affinity with a relevant high-affinity component, and this pattern differed between the two reference molecular strata. One subset showed a compact high-affinity profile and higher mean Wang relatedness than expected under random reassignment, whereas the other was dominated by comparisons with no detectable positive molecular affinity. Importantly, the five-locus SSR framework is interpreted here as an operational, locally validated decision-support tool rather than as genome-wide or pedigree-level relatedness inference. These findings suggest that reduced SSR-derived molecular-affinity information can complement phenotypic, physiological and clonal evaluations by providing redundancy context for line retention, clonal advancement, and parental-diversification decisions in tropical stevia breeding.

## 1. Introduction

*Stevia rebaudiana* Bertoni is a strategically important perennial crop because its leaves accumulate steviol glycosides, a group of high-intensity, non-caloric sweeteners of growing relevance to the food and beverage industry [1,2]. However, the breeding significance of stevia extends well beyond sweetener chemistry alone. Sustainable crop improvement in this species also depends on increasing leaf productivity, improving agronomic stability, broadening adaptation to tropical environments, and reducing the vulnerability associated with a narrow cultivated base [3,4]. This challenge is especially acute in modern stevia cultivation, where commercial expansion has relied heavily on a limited number of clonally propagated elite materials, among which cv. ‘Morita II’ has long occupied a dominant position because of its favourable rebaudioside A profile and agronomic acceptability. Although this cultivar has contributed greatly to crop standardisation, its widespread use has also intensified concerns about reduced adaptive capacity, genetic erosion, and restricted long-term breeding progress [5,6,7].

These concerns are particularly relevant under tropical breeding conditions, where environmental heterogeneity increases the need for differentiated and resilient plant material. In Colombia, previous field evaluations of cv. ‘Morita II’ across contrasting regions showed that edaphoclimatic conditions can differentially affect dry-leaf yield, stevioside, rebaudioside A and the RebA/Stv ratio, reinforcing the need to identify materials with stable agronomic and quality performance under tropical environments [8]. In this context, the breeding programme developed at the Universidad de Córdoba was designed precisely to recover useful variation from a ‘Morita II’-derived segregating population within an integrated improvement framework. Earlier work within this programme demonstrated that the population was not phenotypically uniform and retained biologically meaningful variation in plant architecture, vegetative growth, and phenology under tropical field conditions, thereby supporting the identification of contrasting ideotype candidates within an elite-derived background [9]. In practical terms, these results showed that a restricted founder history does not preclude the persistence of agronomically useful internal variation, provided that such variation is structured and interpreted within a breeding-oriented framework [7,9].

The molecular context of stevia improvement has also evolved substantially in recent years. High-quality genome assemblies and broader interest in precision breeding have strengthened the biological framework for stevia improvement and opened new opportunities for integrating functional genomics with applied selection strategies [7,10,11].

Nevertheless, in breeding programmes operating under resource constraints, reduced SSR panels remain highly valuable when the objective is not exhaustive genome-wide characterisation, but robust discrimination among closely related lines, redundancy detection, programme-level traceability, and operational germplasm management within a narrow elite background. In *Stevia rebaudiana*, molecular-marker approaches, including SSR, EST-SSR, and genic-SSR resources, have been used to classify genetic diversity, support genotype discrimination, assist breeding-oriented germplasm organisation and, more recently, contribute to linkage mapping and QTL detection for steviol glycoside-related traits, reinforcing their applied value beyond identity control alone [5,12,13,14,15].

Within the Universidad de Córdoba programme, the curated five-locus SSR dataset analysed in the present study was generated from 85 genotypes retained after marker optimisation and data curation. This dataset provided an operational molecular framework for assessing line discrimination, internal molecular-affinity structure, and redundancy patterns within the ‘Morita II’-derived breeding collection. From a breeding standpoint, the relevant question was not only whether molecular variation existed within the collection, but whether promising lines were distributed across sufficiently distinct molecular-affinity contexts to support redundancy-aware retention and effective parental diversification.

This distinction is especially important in clonally exploited and founder-restricted breeding systems. A line may appear agronomically promising and even belong to a distinct molecular subset yet still remain embedded within a high-affinity molecular neighbourhood, thereby offering limited value for broadening the breeding base. In practical terms, breeding decisions cannot rely solely on visible superiority or on general molecular subdivision; they must also consider whether elite candidates occupy compact, redundancy-prone subsets or broader, less-redundant molecular contexts. This is consistent with the broader logic of quantitative breeding, where the value of selection depends not only on identifying superior individuals, but also on organising genetic resources in a way that avoids unnecessary redundancy and preserves useful variation across selection cycles [16]. In stevia specifically, SSR-based fingerprinting and relatedness analyses have already been shown to reveal strong molecular affinity among genotypes and to support the need for incorporating less-redundant germplasm into breeding-oriented collections [12].

Relatedness-based analysis therefore provides a particularly useful framework for extending the value of molecular data beyond structure and identity control. Marker-based pairwise relatedness estimation has long been used to describe relative genetic affinity when explicit pedigree information is unavailable or incomplete, although its interpretation depends on marker density, allele-frequency structure, and the biological context of the analysed population [17,18,19,20]. In this context, pairwise Wang relatedness estimation offers an interpretable view of internal SSR-derived molecular affinity, while hierarchical organisation of molecular affinity can reveal whether promising lines are concentrated within compact, redundancy-prone subsets or distributed across broader molecular reservoirs. When these patterns are evaluated against random expectation, they provide a practical basis for distinguishing between materials that are promising but potentially redundant and those that may contribute more effectively to parental diversification and less-redundant line retention.

Accordingly, the present study analysed a curated five-locus SSR dataset from a ‘Morita II’-derived breeding collection to determine whether SSR-derived molecular-affinity patterns can provide operational support for redundancy screening and breeding-context interpretation. Specifically, this study aimed to: (i) quantify the global and group-specific distribution of SSR-derived molecular-affinity classes; (ii) compare whether the two reference molecular partitions differed in their internal molecular-affinity profiles; (iii) describe hierarchical patterns of Wang-derived molecular affinity among genotypes; and (iv) assess whether observed mean within-group relatedness exceeded random expectation under permutation testing. By addressing these objectives, this work positions a reduced SSR panel as a complementary operational tool for redundancy-aware line management, rather than as a genome-wide or pedigree-level framework.

## 2. Results

### 2.1. Performance of the Retained Five-Locus SSR Panel

To justify the analytical use of the reduced SSR panel, locus-level marker performance was summarised from the curated 85-genotype matrix (Table 1). The five retained loci jointly produced 45 alleles, with allele numbers ranging from three to 16 per locus and PIC values ranging from 0.0345 to 0.7686. Matrix completeness was high, with 420 valid locus–genotype scores out of 425 possible combinations, corresponding to 98.8% completeness. Two loci, SUGMS28 and gi18465673, showed particularly high informativeness, whereas gi18465444 provided moderate discrimination and gi16949765 and SUGMS43 contributed lower but reproducible single-locus information. Accordingly, the retained panel was interpreted as a locally validated operational tool for genotype discrimination and redundancy screening within this specific ‘Morita II’-derived breeding collection, rather than as a genome-wide or pedigree-level relatedness framework.

### 2.2. Global Distribution of SSR-Derived Molecular-Affinity Classes

Pairwise relatedness analysis based on the Wang estimator revealed heterogeneous SSR-derived molecular-affinity patterns within the curated 85-genotype dataset. At the global collection level, 1981 of the 3570 pairwise comparisons (55.5%) showed no detectable positive molecular affinity (NR), whereas 628 comparisons (17.6%) were classified as intermediate molecular affinity (IMA) and 961 comparisons (26.9%) were classified as high molecular affinity (HMA) (Table 2). These classes are used here as operational SSR-derived molecular-affinity categories and should not be interpreted as definitive genealogical or pedigree assignments. This distribution showed that the breeding collection retained a substantial proportion of comparisons with no detectable positive molecular affinity, while also containing a relevant high-affinity component.

**Figure 1:**
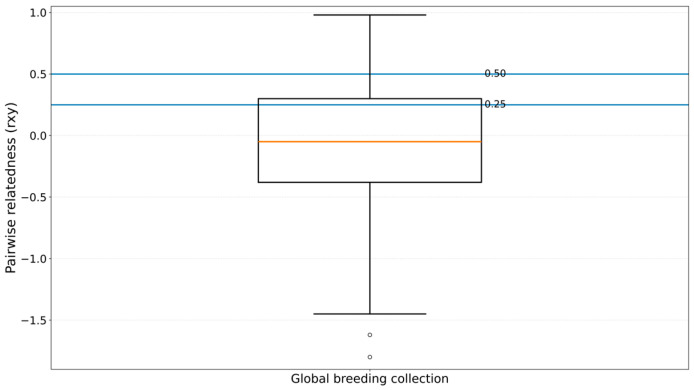
Distribution of pairwise Wang relatedness values (*r_xy_*) in the global *Stevia rebaudiana* breeding collection. The distribution shows the frequency of pairwise comparisons across the curated ‘Morita II’-derived SSR dataset and provides the basis for classifying comparisons into no detectable positive molecular affinity (NR), intermediate molecular affinity (IMA), and high molecular affinity (HMA). The coloured horizontal reference lines indicate *r_xy_* = 0.25, the operational threshold separating IMA from HMA, and *r_xy_* = 0.50, included only as an additional visual reference for stronger positive pairwise affinity. The NR/positive-affinity boundary remains defined as *r_xy_* ≤ 0, as described in the text.

The distribution of *r_xy_* values (Figure 1) further showed that Wang relatedness estimates were not concentrated around a single affinity class but extended across a broad range of SSR-derived molecular-affinity values. This pattern indicates that the collection cannot be interpreted simply as either broadly diverse or uniformly redundant. Instead, it contained both comparisons with no detectable positive molecular affinity and a substantial high-affinity component.

Together, these global molecular-affinity patterns provided the basis for the subsequent group-wise comparison, in which the two reference molecular strata were examined to determine whether their internal distributions of NR, IMA, and HMA comparisons differed within the same SSR dataset. This comparison was used to describe contrasting molecular-affinity profiles, rather than to validate the reference groups as independent biological strata.

### 2.3. Group-Wise Distribution of SSR-Derived Molecular-Affinity Classes

**Figure 2:**
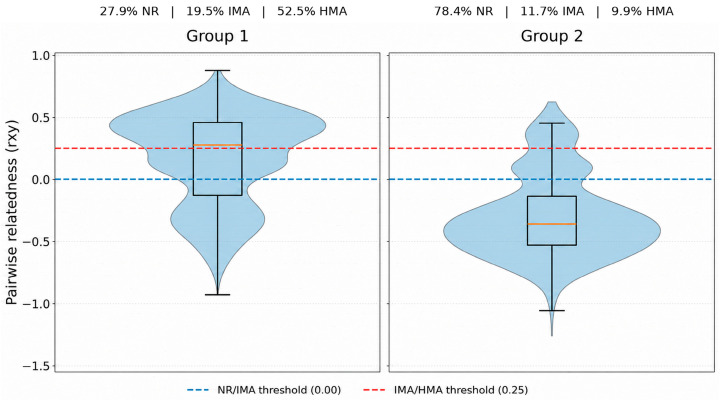
Relative distribution of pairwise SSR-derived molecular-affinity categories in the two reference molecular groups used in the present study for the *Stevia rebaudiana* breeding collection based on the Wang estimator. NR, no detectable positive molecular affinity; IMA, intermediate molecular affinity; HMA, high molecular affinity. In each boxplot, the orange horizontal line indicates the median pairwise Wang relatedness value. The dashed horizontal lines indicate the operational thresholds used for molecular-affinity classification: *r_xy_* = 0 separates NR from positive molecular affinity, whereas *r_xy_* = 0.25 separates IMA from HMA.

When pairwise Wang relatedness was examined separately within the two reference molecular strata, a clear contrast emerged in their internal SSR-derived molecular-affinity profiles. Group 1 showed 415 comparisons with no detectable positive molecular affinity out of 1485 total within-group pairwise contrasts (27.9%), whereas 290 comparisons (19.5%) were classified as intermediate molecular affinity and 780 comparisons (52.5%) as high molecular affinity. By contrast, Group 2 showed 341 comparisons with no detectable positive molecular affinity out of 435 total within-group contrasts (78.4%), 51 intermediate-affinity comparisons (11.7%), and only 43 high-affinity comparisons (9.9%). These results indicate that the two reference molecular strata differed in the distribution of operational molecular-affinity classes (Figure 2). The two reference groups are used here as analytical partitions for comparing internal molecular-affinity profiles within the curated SSR dataset, rather than as fixed biological strata or independently validated pedigree groups. Therefore, the purpose of the group-wise comparison was not to validate the existence of molecular groups, but to determine whether these reference partitions captured contrasting internal molecular-affinity profiles within the curated SSR dataset [12,16].

Accordingly, the observed contrast between Group 1 and Group 2 should be understood as an internal comparison of molecular-affinity profiles within the same SSR dataset, not as external corroboration of population structure.

This group-wise contrast showed that the two reference molecular strata differed not only in the presence of molecular subdivision, but also in the distribution of SSR-derived molecular-affinity classes. Group 1 was characterised by a higher proportion of HMA comparisons, whereas Group 2 was characterised by a predominance of NR comparisons.

### 2.4. UPGMA Clustering Describes Hierarchical Patterns of Wang-Derived Molecular Affinity

**Figure 3:**
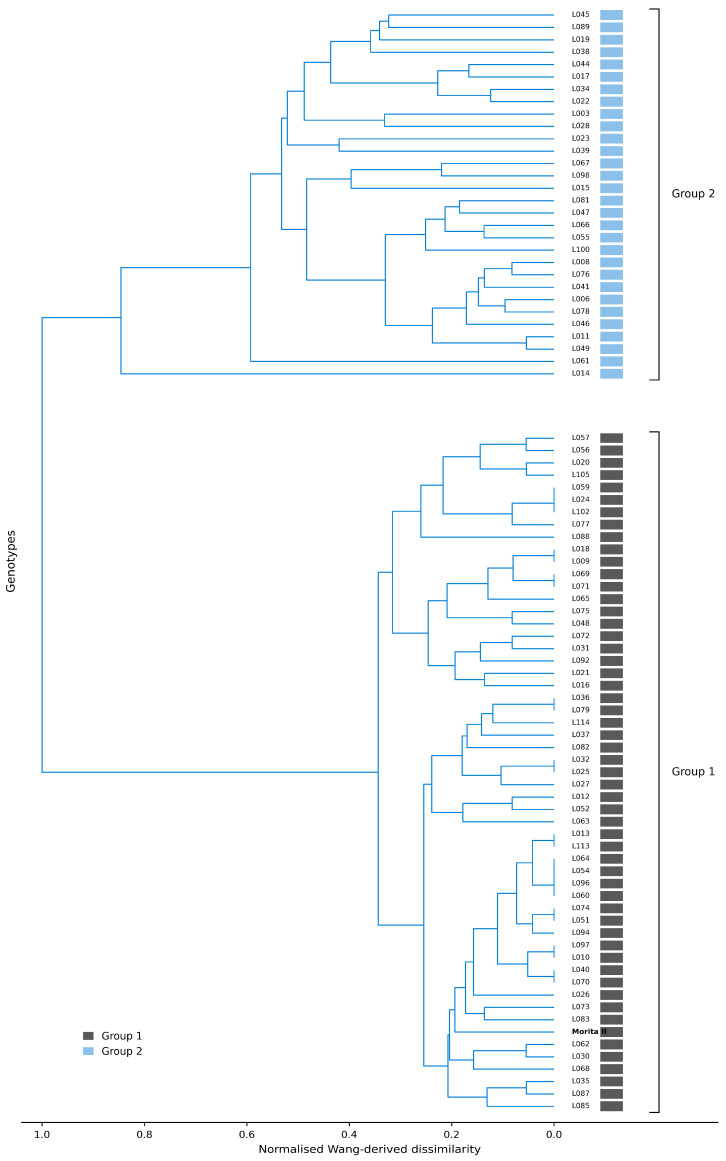
UPGMA dendrogram of 85 *Stevia rebaudiana* genotypes constructed from the Wang-derived dissimilarity matrix based on the Wang relatedness estimator (*r_xy_*). The dendrogram is organised according to the two reference molecular groups used in the present study and descriptively illustrates the higher molecular-affinity profile of Group 1 and the broader dispersion of Group 2.

The UPGMA dendrogram constructed from the Wang-derived dissimilarity matrix (Figure 3) provided an exploratory and descriptive visualisation of molecular affinity within the curated *Stevia rebaudiana* breeding collection. The resulting topology was interpreted only as a representation of relative Wang-derived affinity among genotypes, not as a population-structure analysis, phylogeny, or pedigree reconstruction.

A descriptive contrast in hierarchical organisation was observed between the two subsets. Group 1 formed a more cohesive branch of the dendrogram, consistent with its higher frequency of HMA comparisons. By contrast, Group 2 was distributed more broadly across the tree, consistent with its higher proportion of NR comparisons. This pattern should be interpreted as descriptive support for contrasting molecular-affinity contexts, not as independent confirmation of biological structure [16].

In relation to genotypes of direct programme interest, the control genotype ‘Morita II’ and the elite-relevant lines L020 and L102 were located within Group 1 and in relatively close positions within the dendrogram. These materials had previously been identified as genotypes of direct programme interest through phenotypic, physiological, and clonal evaluation [9,21,22]. Their placement within the more cohesive molecular neighbourhood of Group 1 indicated that these previously identified elite-relevant materials occurred within the subset showing the highest proportion of HMA comparisons. This result was used only as a descriptive molecular context for their relative position within the reduced SSR dataset.

Thus, the UPGMA analysis provided a descriptive representation of the collection based on relative molecular affinity. Within the limits of the reduced SSR panel and the absence of branch-support analysis, the dendrogram was used only to describe relative molecular-affinity patterns and the position of previously identified elite-relevant materials, rather than as an independent endpoint of molecular characterisation [12,16].

### 2.5. Permutation Analysis Evaluates Observed Mean Wang Relatedness Patterns

Permutation analysis was used to evaluate whether observed mean Wang relatedness within the reference molecular strata exceeded random expectation under group-size-preserving reassignment. At the global level, the observed statistic showed upper-tailed departure from the empirical null expectation generated under random reassignment (*p* = 0.034), suggesting that the observed distribution of Wang relatedness was not fully explained by arbitrary group composition.

The group-wise permutation results, integrated with the molecular-affinity class profiles in Table 3, indicated that this pattern was mainly associated with Group 1. This subset showed upper-tailed evidence of higher mean Wang relatedness than expected under random reassignment (*p* = 0.002), consistent with its compact high-affinity profile. By contrast, Group 2 did not show upper-tailed evidence of excess mean Wang relatedness (*p* = 0.948). Therefore, the non-significant result for Group 2 was interpreted only as absence of detectable enrichment of internal molecular affinity under the tested null model, not as positive statistical evidence of diversification potential.

**Figure 4:**
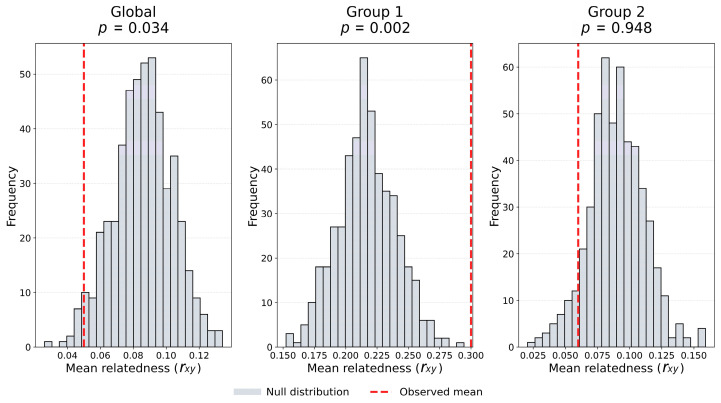
Permutation-based support for observed mean relatedness patterns in the two reference molecular groups used in the present study for the *Stevia rebaudiana* breeding collection.

Within the limits of the reduced five-locus SSR dataset and the 500-permutation procedure, these results indicated contrasting mean Wang relatedness patterns between the two reference molecular strata (Figure 4) [12,16].

## 3. Discussion

### 3.1. SSR-Derived Molecular Affinity as Operational Support for Redundancy-Aware Breeding Decisions

The present study suggests that SSR-derived molecular-affinity information can provide useful redundancy context for interpreting previously identified elite-relevant lines within a ‘Morita II’-derived breeding collection. The more descriptive presentation of these results is interpreted here within a breeding-oriented framework, where the contrasting NR, IMA, and HMA profiles provide operational meaning for redundancy-aware line management. This operational layer is especially relevant in clonally exploited and founder-restricted breeding programmes, where the practical challenge is not only to detect molecular variation, but also to determine whether promising materials occupy sufficiently distinct molecular-affinity contexts to avoid reinforcing redundancy during line retention. In this sense, the present work moves beyond broad molecular subdivision as a descriptive endpoint and shows that pairwise Wang relatedness, descriptive hierarchical clustering, and permutation-based assessment can refine the operational interpretation of elite-relevant materials within a breeding collection [16,23,24,25].

Importantly, the comparison between Group 1 and Group 2 should not be interpreted as an attempt to revalidate population structure using relatedness estimates. Instead, these groups were used as reference molecular partitions to evaluate whether previously defined SSR-based subsets represented contrasting redundancy contexts for breeding decision-making. This distinction is essential because the same SSR dataset was used to define the reference partitions and to estimate Wang relatedness; therefore, the group-wise comparison is interpreted as a within-dataset redundancy assessment rather than as independent confirmation of biological structure.

This interpretation is especially relevant in *Stevia rebaudiana*, where commercial cultivation has long depended on a narrow cultivated base centred on a few clonally propagated materials, particularly cv. ‘Morita II’. Previous studies have highlighted restricted genetic diversity in cultivated stevia germplasm, the need to broaden the use of plant genetic resources, and the importance of integrating molecular information into breeding-oriented germplasm management [3,5,6,7,12]. Under such conditions, the detection of molecular groups is useful, but not sufficient. A line may belong to a differentiated subset and still remain embedded within a compact high-affinity molecular neighbourhood. From a breeding perspective, this matters because retaining multiple elite-looking lines from the same high-affinity background may duplicate molecular redundancy rather than broaden the useful diversity of the programme. The present study addresses this gap by showing that SSR-derived molecular-affinity patterns can provide a more decision-oriented interpretation of molecular data than subdivision alone.

The breeding relevance of this distinction becomes clearer when the present results are interpreted within the wider logic of the programme. Earlier phases had already shown that the ‘Morita II’-derived population retained meaningful phenotypic variation and that some lines, particularly L020 and L102, expressed promising physiological, productive and clonal attributes under tropical conditions [9,21,22]. However, those previous findings did not resolve whether such elite-relevant materials also contributed strategically useful molecular distinctiveness in terms of redundancy control. The present study adds this missing layer by showing that elite performance and low molecular redundancy are not equivalent concepts. A genotype may be agronomically valuable and still occur within a compact high-affinity subset whose repeated retention could limit the broadening of the breeding base.

From this perspective, SSR-derived molecular-affinity information should be understood as a complementary operational filter for breeding decisions. It does not replace phenotypic evaluation, physiological screening, or clonal validation, but it helps contextualise them by distinguishing between elite-relevant materials embedded in redundancy-sensitive high-affinity contexts and those positioned in broader molecular backgrounds. In practical terms, this shifts the role of SSR-based analysis from identity control and molecular subdivision alone to a more operational framework for redundancy-aware line management, particularly in tropical stevia improvement where agronomic performance, glycoside quality, adaptation, and useful genetic variation must be considered jointly [3,14,23].

### 3.2. Contrasting Redundancy Profiles and Their Implications for Clonal Advancement Versus Parental Diversification

A central contribution of the present study is the demonstration that the two reference molecular subsets used in the present analysis do not represent equivalent breeding contexts. Although both belong to the same founder-restricted programme, they differed markedly in their internal SSR-derived molecular-affinity profiles. Group 1 showed a higher frequency of HMA comparisons, with more than half of its pairwise comparisons falling within the high molecular-affinity category, whereas Group 2 was dominated by NR comparisons and showed only a small fraction of HMA contrasts. This distinction is not trivial. In breeding terms, it means that the two subsets should not be interpreted simply as alternative molecular groups, but as contrasting redundancy contexts with different implications for line retention, clonal advancement, and parental diversification. This interpretation is consistent with the need in stevia breeding to combine selection for yield and glycoside quality with the maintenance of genetic variability for future improvement [3,23].

The interpretation of Group 1 as a compact high-affinity subset is further supported by the descriptive hierarchical pattern and the permutation-based assessment of mean Wang relatedness. Its more cohesive organisation in the UPGMA dendrogram and its higher mean Wang relatedness relative to random expectation indicate that this subset concentrates a stronger molecular-affinity signal than would be expected under arbitrary group composition. In practical terms, this means that the repeated retention of multiple elite-relevant candidates from Group 1 may increase the risk of molecular redundancy, even when those candidates differ phenotypically or perform well agronomically. Under a short-term deployment perspective, such a subset may still be highly useful for clonal advancement, especially when individual genotypes combine strong field performance with verified identity. However, from a longer-term breeding perspective, retaining too many lines from the same compact high-affinity background without additional filtering may reinforce a narrower molecular base rather than expand the effective diversity of the programme.

By contrast, Group 2 represents a broader molecular background within the limits of the reduced SSR dataset. Its high proportion of NR comparisons, together with the absence of upper-tailed evidence for excess mean Wang relatedness, suggests that this subset does not show detectable enrichment of internal molecular affinity under the tested null model. In practical breeding terms, this makes Group 2 useful as a reservoir of less-redundant molecular backgrounds for parental diversification and for the selection of candidates whose retention is less likely to duplicate the same internal affinity pattern. This does not imply that all lines in Group 2 are automatically superior or that Group 1 should be deprioritised. Rather, it indicates that the strategic value of each subset depends on the breeding objective and must be interpreted jointly with phenotypic, physiological, biochemical, and clonal performance data: one subset is more compatible with clonal advancement within a compact elite-relevant context, whereas the other offers greater scope for broadening the molecular base in future selection and recombination cycles. This interpretation is consistent with stevia breeding frameworks that emphasise simultaneous improvement of leaf yield, steviol glycoside composition, environmental adaptation, and parent selection based on useful genetic divergence [3,14,23].

This distinction becomes especially relevant when interpreted alongside previous phases of the breeding programme. Some of the most relevant elite-relevant candidates identified through phenotypic, physiological, and clonal evaluation, including L020 and L102, were located within the more cohesive molecular neighbourhood represented by Group 1 [9,21,22]. This result does not undermine their value. On the contrary, it clarifies the breeding context in which that value should be deployed. These lines may remain highly valuable for clonal multiplication, short-term advancement, and benchmark comparison against ‘Morita II’, but their use as repeated sources of selection or as central contributors to programme diversification should be accompanied by explicit redundancy control. In this sense, the present study refines the meaning of elite relevance by showing that agronomic merit and low molecular redundancy are related but not interchangeable dimensions of breeding value.

Taken together, the contrasting molecular-affinity profiles support a two-track interpretation within the ‘Morita II’-derived programme. Compact high-affinity subsets may be useful for short-term clonal advancement after redundancy filtering, whereas broader molecular backgrounds dominated by NR comparisons may offer greater value for parental diversification and future recombination.

This distinction provides a practical decision rule: high-performing lines from compact high-affinity subsets should be retained cautiously after redundancy filtering, whereas promising lines from broader molecular backgrounds may receive greater consideration for diversification-oriented breeding. Thus, Group 1 should not be interpreted as inferior, but as a redundancy-sensitive elite-relevant context; likewise, Group 2 should not be interpreted as automatically superior, but as a broader molecular reservoir for future breeding decisions [3,4,5,6,7].

### 3.3. Elite Performance and Low Molecular Redundancy Are Not Equivalent

A particularly important implication of the present results is that elite agronomic or physiological performance does not necessarily coincide with low molecular redundancy. This distinction is central to breeding decisions in founder-restricted systems, because a genotype may be highly valuable in productive terms and yet still contribute little to broadening the breeding base if it is embedded within a compact high-affinity molecular background. In other words, elite performance and strategic molecular distinctiveness are related dimensions of breeding value, but they are not interchangeable. The present study makes this distinction explicit by showing that genotypes of recognised programme relevance, including L020, L102, and the commercial benchmark ‘Morita II’, are positioned within the more cohesive molecular-affinity context of Group 1 rather than within the broader NR-dominated molecular background represented by Group 2, according to the reduced SSR dataset analysed here.

This result is especially meaningful when viewed against the earlier stages of the breeding programme. Published studies from the same breeding programme had already shown that L020 and L102 were among the most promising genotypes in terms of physiological performance, productive behaviour, and clonal usability under tropical conditions, while ‘Morita II’ remained the agronomic and industrial reference point of the programme [7,21,22]. The present SSR-derived molecular-affinity analysis does not contradict those findings; rather, it qualifies their molecular interpretation. The fact that these materials occur within the more cohesive molecular-affinity subset indicates that their value for clonal deployment or short-term advancement may be high, while their value for broadening the internal molecular base of the breeding programme may be more limited if they are repeatedly retained alongside closely affine alternatives. This distinction is also relevant because clonal deployment in stevia increasingly requires not only multiplication efficiency, but also attention to genetic stability and steviol glycoside-related performance in propagated or regenerated materials [22,26].

In practical breeding terms, this means that the retention of elite-relevant candidates should not be treated as a single decision category. A genotype such as L020 or L102 may be highly desirable for clonal multiplication, benchmark comparison or direct agronomic deployment, but that does not imply that it should automatically occupy a central role in diversification-oriented breeding decisions. When multiple promising genotypes are concentrated within the same compact high-affinity molecular background, their combined retention may increase redundancy even if each one performs well individually. Under such conditions, molecular context becomes essential for distinguishing between genotypes that are elite-relevant in a deployment sense and those that are more informative in a diversification sense. This distinction is especially relevant in *Stevia rebaudiana*, where the improvement challenge is not only to identify superior materials, but also to prevent the recurrent recycling of a narrow molecular background around dominant or widely used cultivars. This concern is consistent with previous reports of restricted genetic diversity in cultivated stevia germplasm and with recent calls to broaden, conserve, and strategically organise plant genetic resources for breeding [3,4,5,6,7].

From this perspective, the placement of ‘Morita II’, L020, and L102 within Group 1 should be interpreted as strategically informative rather than restrictive. It suggests that these materials define a strong elite-relevant nucleus within the programme, but also that their use must be consciously balanced against the need to prevent redundancy accumulation across future breeding cycles. The role of SSR-derived molecular-affinity information is therefore not to downgrade high-performing lines, but to contextualise their use. In a practical decision framework, some elite-relevant genotypes may be retained primarily for clonal advancement and immediate agronomic exploitation, whereas others may be prioritised for their capacity to expand the programme’s molecular breadth. The present study thus supports a more nuanced concept of elite-relevant line value, in which agronomic excellence must be interpreted jointly with redundancy risk and with the longer-term objective of sustaining useful diversity within the breeding pipeline. This perspective is consistent with stevia breeding approaches that recognise genetic variability, parent selection, and the integration of agronomic and biochemical traits as central elements for long-term cultivar improvement [3,7,23].

### 3.4. Scope, Limitations, and Applied Value of a Reduced SSR-Based Molecular-Affinity Framework

The present study should be interpreted within the analytical scope of a reduced, locally validated SSR panel applied to a single ‘Morita II’-derived breeding collection. This design was sufficient to detect operationally useful contrasts in molecular-affinity profiles, redundancy patterns, and hierarchical affinity structure within the curated 85-genotype dataset, but it does not provide genome-wide resolution and should not be interpreted as equivalent to formal pedigree reconstruction or high-density genomic relatedness inference. Accordingly, the molecular-affinity classes used here are best understood as operational indicators of relative SSR-derived affinity rather than as definitive genealogical or pedigree assignments. This limitation is especially important in a founder-restricted crop such as *Stevia rebaudiana*, where a reduced marker set can still be informative for programme-level decisions, yet cannot capture the full genomic complexity of the breeding population. More broadly, marker-based relatedness estimates are known to be sensitive to marker number, allele-frequency distribution, and population structure, which reinforces the need to interpret SSR-derived molecular-affinity classes as operational signals rather than definitive pedigree assignments [16,17,18,20].

A second limitation is that the present framework was developed within a single breeding context centred on a ‘Morita II’-derived background. The molecular-affinity structure described here should therefore be interpreted as specific to this programme and should not be extrapolated uncritically to all cultivated stevia germplasm. Even so, this contextual specificity is also one of the study’s strengths. The objective was not to produce a universal population-genetic survey, but to extract decision-relevant molecular information from the curated collection analysed in the present study, already embedded in a real breeding pipeline. In that sense, the value of the reduced SSR panel lies precisely in its local validation and operational usefulness within a defined programme, where line distinctiveness, redundancy control and clonal management are immediate practical concerns rather than abstract genetic descriptors. This interpretation is consistent with the broader use of molecular markers to rationalise germplasm collections, detect redundant accessions, support core-collection development, and improve the efficiency of genetic-resource management [5,16,25,27,28,29].

These limitations do not diminish the applied value of the framework. On the contrary, they clarify the scale at which the present results are most useful. In applied breeding programmes operating under logistical and financial constraints, reduced SSR panels remain valuable when the goal is to discriminate closely related materials, organise breeding collections, and support strategic line retention. This is particularly relevant in stevia, where the transition towards more advanced genomic resources is already underway, but where programme-level management decisions still require robust, feasible, and interpretable marker systems.

Recent genomic, epigenomic, multi-omics, and marker-assisted advances, including chromosome-scale assemblies, SNP-based linkage mapping, QTL mapping for steviol glycoside-related traits, and more detailed insight into the regulation of steviol glycoside biosynthesis, clearly expand the long-term prospects for stevia improvement. However, they do not eliminate the immediate usefulness of operational marker frameworks such as the one employed here, particularly in breeding programmes where feasible, interpretable, and locally validated tools are still required for day-to-day germplasm management [7,10,11,14,30,31,32,33].

From this perspective, the reduced SSR-based molecular-affinity framework presented here should be understood as a bridge between classical germplasm management and future high-resolution breeding tools. It is informative enough to identify compact high-affinity contexts, distinguish broader molecular backgrounds, and refine redundancy-aware line management within the present collection. At the same time, its interpretation remains dependent on integration with the phenotypic, physiological, productive, and clonal evidence already generated in other phases of the programme. Thus, the main applied contribution of the present study is not to provide an exhaustive molecular description of the breeding population, but to demonstrate that even a reduced and carefully curated SSR dataset can yield breeding-relevant insight when interpreted through the lens of molecular affinity and redundancy-aware line management. This interpretation is consistent with recent efforts in *Stevia rebaudiana* to integrate elite-genotype evaluation, descriptor-based discrimination, and clustering approaches as practical tools for organising breeding-relevant variation [9,21,22,34].

### 3.5. Strategic Synthesis: SSR-Derived Molecular Affinity as a Complementary Layer for Redundancy-Aware Breeding

Taken together, the present results support a breeding-oriented reinterpretation of the molecular component of the ‘Morita II’-derived breeding programme. The main contribution of this study is not to redefine population structure in *Stevia rebaudiana*, but to show how a reduced, locally validated SSR dataset can be translated into practical decisions for redundancy control, clonal advancement, and parental diversification within a founder-restricted breeding programme. In this sense, SSR-derived molecular-affinity information adds an operational level of meaning that broad molecular subdivision alone could not provide. By distinguishing between compact high-affinity contexts and broader molecular backgrounds dominated by NR comparisons, the present study shows that molecular analysis can move beyond identity control and traceability to support more explicit, redundancy-aware decisions on line retention, clonal advancement, and parental diversification. This interpretation is consistent with the broader role of molecular markers in germplasm rationalisation, redundancy reduction, core-collection development, and strategic use of plant genetic resources for breeding [3,12,16,25,27,28].

This strategic perspective is especially relevant in founder-restricted crops such as *Stevia rebaudiana*, where improvement programmes must often balance immediate commercial utility with the longer-term need to avoid repeated concentration of the same molecular background. Within the present programme, the combination of phenotypic structuring, physiological and productive evaluation, clonal propagation, and SSR-derived molecular-affinity interpretation now allows a more complete breeding logic to emerge. This integrated view is also consistent with Colombian evidence showing that dry-leaf yield and glycoside-quality traits in ‘Morita II’ vary across environments, as well as with recent descriptor-based approaches for organising elite stevia genotypes. Under this framework, line value is not defined by a single dimension, but by the intersection of agronomic promise, functional performance, clonal usability, environmental response, stability of propagated material, steviol glycoside-related performance, and relative molecular position within the collection [8,9,21,22,26,34].

From this perspective, the practical message of the present study is clear. A ‘Morita II’-derived collection may still contain highly valuable elite-relevant candidates, but those candidates are not all equivalent in terms of their contribution to future breeding steps. Some genotypes may be suitable for clonal advancement within a compact elite-relevant background, whereas others may be more important because they occupy broader molecular backgrounds and therefore contribute more effectively to diversification-oriented breeding. In this sense, the present study does not propose replacing phenotypic and agronomic selection with molecular filtering; rather, it proposes refining line-management decisions through a more explicit consideration of redundancy risk. Such an approach is especially valuable in tropical stevia improvement, where the challenge is not only to identify good performers, but also to organise them in a way that sustains useful diversity across cycles of selection and deployment. This need is consistent with previous and recent analyses highlighting that sustainable stevia improvement requires not only the optimisation of promising breeding materials, but also broader use, conservation, molecular characterisation, and strategic organisation of plant genetic resources [3,4,7,35].

Accordingly, SSR-derived molecular-affinity information should be understood as a complementary operational layer that connects molecular position with practical line-management decisions. It helps distinguish elite-relevant materials embedded within compact high-affinity contexts from those occupying broader molecular backgrounds.

This integrated logic is likely to be especially useful for programmes working with narrow founder backgrounds, where elite performance alone is not sufficient to determine long-term breeding value. A genotype may be highly useful for clonal deployment but less informative for diversification if it belongs to a compact high-affinity subset. Conversely, a genotype with acceptable agronomic performance but lower molecular redundancy may be strategically valuable for future recombination. Therefore, the main contribution of the present framework is to support redundancy-aware decisions that balance immediate deployment of promising clonal materials with the long-term need to sustain useful genetic variation across breeding cycles.

This perspective is particularly relevant for tropical stevia breeding because previous Colombian evaluations of ‘Morita II’ demonstrated that dry-leaf yield and glycoside quality traits vary across environments, indicating that line management should integrate molecular position with agronomic adaptation and leaf-quality performance [8].

### 3.6. Breeding Implications for Redundancy-Aware Line Management

Taken together, the global molecular-affinity profile, the contrast between group-specific molecular-affinity patterns, the UPGMA hierarchy, and the permutation-based assessment indicate that redundancy-aware line management in this ‘Morita II’-derived breeding collection cannot rely solely on agronomic promise or broad molecular distinctiveness. Rather, the results show that candidate lines should also be interpreted in light of their SSR-derived molecular-affinity context, because the same breeding collection contains both a substantial proportion of comparisons with no detectable positive molecular affinity and a compact high-affinity subset. In this sense, molecular value is conditioned not only by whether a line is promising, but also by whether its retention is likely to reinforce redundancy within the selected set or contribute to broadening the breeding base. This distinction is particularly relevant in stevia breeding, where selection must reconcile leaf yield, glycoside quality, adaptation, and the maintenance of useful genetic variation [3,23].

From this perspective, Group 1 should be interpreted as a compact high-affinity subset requiring particular caution during clonal advancement and line retention. Its enrichment in HMA comparisons, together with its higher mean Wang relatedness relative to random expectation, indicates that the repeated retention of multiple candidates from this subset may reinforce a redundancy-sensitive molecular background rather than expand useful diversity within the programme. By contrast, Group 2 represents a broader molecular background dominated by NR comparisons, in which line retention is less likely to duplicate the same molecular-affinity signal and may therefore offer greater value for parental diversification and subsequent recombination-oriented steps. This distinction is especially relevant in founder-restricted breeding systems, where selection intensity may otherwise favour the repeated accumulation of molecularly similar materials under the appearance of independent superiority [3,5,6,7,23].

This interpretation becomes even more important when considered alongside previous phases of the programme. Earlier work had already shown that the breeding pipeline retained phenotypically structured variation and that some genotypes, particularly L020 and L102, expressed desirable physiological, productive, and clonal attributes under tropical conditions [9,21,22]. The present analysis adds a further layer of interpretation by showing that some of these elite-relevant materials are positioned within the more cohesive molecular-affinity context of Group 1. This does not diminish their strategic value; rather, it refines it. Under the present framework, such genotypes may remain highly valuable for clonal deployment or short-term advancement, but their use should be accompanied by explicit redundancy control if the broader objective is to sustain long-term diversification within the programme.

Accordingly, the present study supports a two-level breeding interpretation of the curated molecular collection. Genotypes located within compact high-affinity subsets may be prioritised where the main objective is clonal advancement of agronomically favourable material, whereas genotypes embedded within broader molecular backgrounds dominated by NR comparisons may be more suitable for parental diversification and for strengthening the adaptive breadth of future breeding cycles. In this sense, SSR-derived molecular-affinity information functions as a complementary decision-support layer that helps reconcile immediate deployment of promising clonal materials with longer-term germplasm management. Rather than simply confirming that the collection is structured, the present analyses show how molecular-affinity information translates SSR-derived data into operational breeding meaning by distinguishing between promising but potentially redundant candidates and those more likely to contribute to diversification-oriented improvement [12,16,23,24,25].

In practical terms, the framework can be applied as a sequential decision-support procedure within this breeding programme. First, candidate lines should be confirmed using reproducible SSR profiles and previously generated phenotypic, physiological and clonal information. Second, pairwise SSR-derived molecular-affinity classes should be used to identify whether elite-relevant materials are embedded within compact HMA-dominated contexts or broader NR-dominated molecular backgrounds. Third, repeated retention of multiple elite-relevant candidates from the same compact high-affinity neighbourhood should be avoided unless justified by clearly superior agronomic or clonal performance. Fourth, less-redundant materials positioned in broader molecular backgrounds should be considered preferentially when the breeding objective is parental diversification or future recombination. These steps are intended for within-programme line management in this ‘Morita II’-derived collection and should be recalibrated before application to unrelated stevia germplasm or higher-resolution genomic datasets.

## 4. Materials and Methods

### 4.1. Plant Material and Analytical Scope of the Molecular Dataset

The present study was conducted within the *Stevia rebaudiana* breeding programme of the Universidad de Córdoba, Montería, Colombia, developed under tropical dry-forest conditions. The broader programme originated from a ‘Morita II’-derived segregating population generated through controlled crossing and subsequent selection within a restricted founder background. The original breeding population comprised 115 progenies, from which 86 progeny-derived genotypes were retained and clonally propagated for advanced evaluation. The commercial cultivar ‘Morita II’, also clonally propagated, was included as a reference control within the broader experimental pipeline. Accordingly, the phenotypic phase of the programme operated with 87 treatments, comprising 86 progeny-derived genotypes plus the control cultivar [9].

For the molecular component of the programme, not all materials from the broader clonal set were carried forward into the final SSR dataset. After marker optimisation, genotyping, and dataset curation, one progeny-derived genotype did not yield a reliable multilocus SSR profile and was therefore excluded from the final molecular matrix. As a result, the curated SSR dataset used in the present study comprised 85 genotypes. This molecular subset should therefore be interpreted as a breeding-oriented, SSR-validated clonal subset derived from the broader ‘Morita II’-based improvement pipeline, rather than as a census of all materials evaluated phenotypically.

Importantly, the objective of the present manuscript is not to provide an exhaustive molecular characterisation of the collection, but to analyse the curated SSR dataset under a distinct breeding-oriented objective: to determine whether the internal SSR-derived molecular-affinity structure of the breeding collection provides decision-support value for redundancy-aware line management, redundancy control, and parental diversification. Under this framework, the analytical scope of the present paper is explicitly centred on the molecular-affinity structure of the curated 85-genotype molecular set and on its breeding implications within the ongoing stevia improvement programme.

### 4.2. SSR Dataset and Reference Strata Used for Molecular-Affinity Analysis

Molecular-affinity analyses were conducted using the final multilocus SSR genotype matrix derived from the five informative loci retained after marker optimisation and curation within the molecular component of the breeding programme. This reduced marker panel was selected because it provided consistent amplification profiles and sufficient discriminatory value for analysing closely related materials within the ‘Morita II’-derived breeding collection. In the present work, this curated multilocus matrix was analysed to quantify internal SSR-derived molecular affinity, examine redundancy patterns, and support breeding-oriented interpretation of line relationships within the collection.

The SSR panel used in this study comprised five loci: SUGMS28, SUGMS43, gi18465444, gi16949765, and gi18465673. These loci were retained after excluding stvia036, which failed to generate reliable amplification products under the tested analytical conditions. The use of SSR loci for stevia genotyping and germplasm discrimination is consistent with previous marker-development and germplasm-characterisation studies in the species, as well as with the broader utility of microsatellites for codominant, reproducible, and highly informative marker-based analysis in plants [12,36,37]. The resulting five-locus matrix should therefore be interpreted as a locally validated, operational marker set designed for line discrimination and germplasm management within this specific breeding collection, rather than as a genome-wide representation of diversity in cultivated stevia. The performance of the retained loci is summarised in Table 1, including allele number, allele-size range, Ho, He, PIC, missing/zero data, and locus-level contribution to genotype discrimination. These metrics were used to define the retained marker set as an operational SSR panel for local molecular-affinity analysis, not as a genome-wide or pedigree-level framework.

Following PCR optimisation, amplification products were initially checked by electrophoresis in 2% agarose gels run at 90 V for 30 min and stained with FloroVue™ Nucleic Acid Gel Stain (SMOBIO Technology, Inc., Hsinchu City, Taiwan). This agarose-gel step was used only as a qualitative verification of PCR amplification and was not used for allele sizing or genotype scoring. For SSR genotyping, fluorescently labelled amplification products were separated by capillary electrophoresis using a SeqStudio Genetic Analyzer (Applied Biosystems, Foster City, CA, USA), with an internal size standard included for fragment sizing. Allele calling and genotype scoring were performed using GeneMarker v3.0 software (SoftGenetics, State College, PA, USA). The resulting allele-size calls were curated to generate the final five-locus SSR genotype matrix used for molecular-affinity analysis. Representative capillary electropherograms of SSR amplification products in selected *Stevia rebaudiana* genotypes are provided as Supplementary Figure S1.

For the purposes of the present study, the curated SSR dataset was organised into two reference molecular strata, hereafter referred to as Group 1 and Group 2. These strata were derived from a previously curated multilocus SSR-based molecular assignment of the same 85-genotype dataset and were retained here exclusively as operational analytical partitions for group-wise molecular-affinity comparisons. Importantly, Group 1 and Group 2 were not treated as fixed biological populations, pedigree groups, or independent taxonomic units. Instead, they were used to examine whether previously defined molecular partitions differed in their internal SSR-derived molecular-affinity profiles within the ‘Morita II’-derived breeding collection. This distinction was essential to avoid conflating two analytical levels: first, the existence of broad molecular partitions within the curated SSR dataset; and second, the internal SSR-derived molecular-affinity profile of each partition. Because both the reference molecular strata and the Wang relatedness estimates were derived from the same five-locus SSR matrix, the group-wise comparison was not interpreted as an independent validation of molecular structure. Rather, it was used as a within-dataset descriptive comparison of molecular-affinity profiles in addition to broad molecular grouping.

This distinction is especially important in a founder-restricted and clonally managed breeding context. In such systems, assignment to a molecular group does not by itself indicate whether a genotype contributes meaningfully to diversification or remains embedded within a compact high-affinity molecular neighbourhood. Accordingly, the reference-strata framework adopted here was intended to support a more operational interpretation of the curated SSR dataset, allowing SSR-derived molecular-affinity patterns to be evaluated both at the global collection level and within each reference molecular subset. In this sense, the value of the SSR matrix in the present study lies not in re-establishing genetic structure, but in providing a stable molecular scaffold for describing SSR-derived molecular-affinity patterns within the curated breeding dataset [16].

### 4.3. Wang Relatedness Estimation and Operational Molecular-Affinity Classification

Pairwise relatedness among genotypes was estimated from the codominant SSR genotype matrix using the Wang estimator (*r_xy_*) [20]. Marker-based relatedness estimation has been widely used when pedigree information is incomplete or unavailable, although estimator performance depends on marker number, allele-frequency structure, and population context, and should therefore be interpreted within an explicitly operational framework [17,18,19,20,38]. The Wang estimator was selected because it provides an interpretable marker-based measure of relative molecular affinity for codominant markers and is therefore useful for breeding collections derived from restricted founder backgrounds. In the present study, it was used as an operational measure of molecular affinity rather than as a formal pedigree reconstruction tool.

All pairwise genotype comparisons were classified into three operational SSR-derived molecular-affinity categories. Comparisons with *r_xy_* ≤ 0 were classified as showing no detectable positive molecular affinity (NR). Comparisons with 0 < *r_xy_* ≤ 0.25 were classified as intermediate molecular affinity (IMA), whereas comparisons with *r_xy_* > 0.25 were classified as high molecular affinity (HMA). These thresholds were used only as operational affinity classes for redundancy-oriented interpretation and should not be understood as definitive genealogical or pedigree assignments. For this reason, sibship or parent–progeny terminology was avoided in the Results and Discussion, except when explaining the conventional rationale behind the thresholds.

Relatedness calculations were performed in R using the related package, which implements several marker-based relatedness estimators for codominant molecular data, including the Wang estimator [39]. Pairwise relatedness was estimated with the Wang function from the curated codominant SSR genotype matrix, and all downstream summaries were generated from the resulting pairwise *r_xy_* matrix.

### 4.4. Group-Wise Comparison of SSR-Derived Molecular-Affinity Profiles

To determine whether the reference molecular strata differed in their internal SSR-derived molecular-affinity profiles, pairwise Wang relatedness categories were summarised separately for the global collection and for each reference group. These strata were used as an analytical framework to evaluate whether each subset represented a distinct redundancy context within the curated SSR dataset. This analysis was not intended to infer independent population structure or to assign definitive ancestry to individual genotypes. Rather, it evaluated whether the two reference partitions differed in the frequency of operational molecular-affinity classes within the curated SSR dataset. For each group, all within-group pairwise comparisons were classified into NR, IMA, and HMA categories using the thresholds described above. The resulting counts and percentages were used to compare the relative contribution of comparisons showing no detectable positive molecular affinity, intermediate molecular affinity, and high molecular affinity within each subset. Under the present analytical framework, a group enriched in HMA comparisons was described as a compact high-affinity context, whereas a group dominated by NR comparisons was described as a broader molecular background within the curated SSR dataset. This comparison allowed the collection to be examined not only in terms of broad molecular subdivision, but also in terms of how SSR-derived molecular-affinity classes were distributed within each subset. In this sense, group-wise molecular-affinity profiles were used to describe differences in the distribution of NR, IMA, and HMA comparisons within the curated SSR dataset, rather than as direct evidence of fixed pedigree structure.

### 4.5. Hierarchical Clustering Based on Wang-Derived Dissimilarity

To describe hierarchical patterns of relative molecular affinity among genotypes, the pairwise Wang relatedness matrix was transformed into a Wang-derived dissimilarity matrix suitable for exploratory hierarchical clustering. Because Wang relatedness values can include negative and positive estimates, *r_xy_* values were transformed before clustering. Dissimilarity between genotypes *i* and *j* was calculated as: *dij* = 1 − *r_xy_ij*, where *r_xy_ij* is the Wang relatedness estimate for a given pair of genotypes. Under this transformation, higher Wang relatedness values produce lower dissimilarity values, whereas lower or negative relatedness values produce higher dissimilarity values. Because Wang relatedness estimates may include negative values, the resulting Wang-derived dissimilarities may exceed 1 in some pairwise comparisons. These values were retained because they represent lower-than-average molecular affinity under the estimator, rather than true negative genealogical relationships. Accordingly, the UPGMA dendrogram was interpreted as an exploratory representation of relative molecular affinity and affinity neighbourhoods, not as a metric phylogeny or formal pedigree reconstruction.

The resulting Wang-derived dissimilarity matrix was used to construct a hierarchical tree using the unweighted pair-group method with arithmetic mean (UPGMA). The dendrogram was interpreted in relation to the two reference molecular strata used in the present study, with emphasis on differences in compactness, dispersion, and neighbourhood-level proximity among genotypes. The purpose of this clustering analysis was not to reconstruct pedigree relationships formally, but to provide a breeding-oriented representation of internal molecular-affinity structure. No branch-support or resampling-based node stability analysis was used; therefore, the dendrogram was interpreted only as a descriptive representation of relative molecular-affinity patterns.

Particular attention was paid to the relative position of genotypes of direct breeding interest, including ‘Morita II’, L020, and L102. Compact clustering was treated as a descriptive indication of stronger relative molecular affinity, whereas broader dispersion was treated as a descriptive indication of lower relative molecular affinity within the reduced SSR dataset. Hierarchical clustering was performed in R using the ape package v5.0, an environment for phylogenetic, and evolutionary analyses in R [40]. The UPGMA tree was constructed from the Wang-derived dissimilarity matrix using the upgma function applied to the corresponding distance object.

### 4.6. Permutation-Based Assessment of Mean Relatedness

To assess whether the observed mean relatedness within each molecular stratum exceeded random expectation, a permutation-based procedure was applied to the pairwise Wang relatedness matrix. For each reference group, the observed statistic corresponded to the mean within-group Wang relatedness calculated from all pairwise comparisons among genotypes assigned to that group. This observed mean was compared with an empirical null distribution generated by randomly reassigning genotypes between groups while preserving the original group sizes. For the global assessment, the observed statistic corresponded to the mean of the group-specific within-group Wang relatedness values, calculated under the observed reference partition and compared with the corresponding empirical null distribution generated under the same group-size-preserving reassignment procedure. This procedure allowed the observed molecular-affinity pattern to be evaluated relative to a random grouping scenario.

A total of 500 permutations were performed. In each permutation, individuals were randomly reassigned to groups of the same size as the observed groups, and the mean within-group relatedness was recalculated. The empirical upper-tailed *p*-value was estimated as the proportion of permutations in which the permuted mean relatedness was equal to or greater than the observed mean, using the observed statistic as the reference value. Given the use of 500 permutations, empirical *p*-values were interpreted as supportive evidence of departure from random expectation rather than as high-resolution inferential probabilities.

This procedure was used to assess whether a given molecular stratum showed evidence of higher internal molecular affinity than expected under random reassignment. Groups showing upper-tailed evidence of higher mean relatedness than the null expectation were described as high-affinity subsets within the tested SSR-derived molecular-affinity framework. Groups whose observed means did not exceed random expectation were interpreted only as showing no detectable enrichment of internal molecular affinity under the tested null model. Because the test was based on 500 permutations and a reduced SSR panel, the results were treated as statistical support for describing mean molecular-affinity patterns, rather than as definitive validation of pedigree structure or independent population structure.

Permutation analyses were conducted in R using a random reassignment procedure with 500 permutations, implemented with support from the vegan package while preserving the original group sizes [41].

### 4.7. Analytical Integration of SSR-Derived Molecular-Affinity Patterns

The outputs from pairwise Wang relatedness estimation, operational molecular-affinity classification, group-wise summaries, UPGMA clustering, and permutation-based assessment were integrated within a predefined analytical framework. This framework was designed to relate SSR-derived molecular-affinity patterns to the previously documented phenotypic, physiological, and clonal evidence generated in other phases of the ‘Morita II’-derived breeding programme [9,21,22].

Molecular-affinity information was therefore treated as a complementary analytical layer, rather than as a replacement for agronomic, physiological, or clonal evaluation.

For analytical consistency, the combined evidence from pairwise molecular-affinity classes, group-wise molecular-affinity profiles, hierarchical clustering, and permutation-based assessment was used to describe two broad molecular contexts within the curated SSR dataset. The first corresponded to genotypes located within compact high-affinity contexts, whereas the second corresponded to genotypes positioned within broader molecular backgrounds dominated by NR comparisons. These molecular contexts were subsequently interpreted in the Discussion in relation to redundancy-aware line management, clonal advancement, and parental diversification [3,23,25].

Previous phases of the programme were used only as contextual information for selecting genotypes of direct programme interest and for interpreting their relative position within the SSR-derived molecular-affinity framework. These included prior reports of structured phenotypic variation in the ‘Morita II’-derived population, the identification of L020 and L102 as agronomically and physiologically promising genotypes, and clonal propagation evidence supporting their practical use under in vitro conditions [9,21,22]. In the present study, these materials were not re-ranked on the basis of SSR data; instead, their relative molecular-affinity context was described within the curated SSR dataset.

Accordingly, the analytical integration focused on three descriptive outputs: molecular-affinity class distribution, relative hierarchical position, and permutation-supported mean relatedness patterns. The breeding implications of these outputs were developed in the Discussion, where they were considered in relation to redundancy control, clonal advancement and parental diversification within a founder-restricted stevia breeding system [16].

## 5. Conclusions

This study supports the use of a reduced, locally validated five-locus SSR panel as an operational tool for describing molecular-affinity and redundancy patterns within a ‘Morita II’-derived *Stevia rebaudiana* breeding collection. Rather than reconstructing pedigree relationships or independently ranking candidate lines, the analysis provides molecular redundancy context for previously identified elite-relevant materials and helps distinguish compact high-affinity subsets from molecular backgrounds dominated by comparisons with no detectable positive molecular affinity. Within these limits, SSR-derived molecular-affinity information can complement phenotypic, physiological, and clonal evaluations by supporting redundancy-aware line retention, clonal advancement, and the identification of less-redundant materials for future breeding decisions.

## Figures and Tables

**Table 1:** Performance summary of the five SSR loci retained for molecular-affinity analysis in the ‘Morita II’-derived *Stevia rebaudiana* breeding collection.

Locus	Valid Genotypes, *n*	Missing/Zero Genotypes, *n*	Number of Alleles	Allele-Size Range (bp)	Ho	He	PIC	Amplification Quality	Contribution to Genotype Discrimination
gi16949765	85	0	3	99–189	0.0353	0.0348	0.0345	Complete scoring; no missing/zero genotypes	Low single-locus informativeness; retained as a reproducible locus contributing marginally to multilocus identity control
SUGMS28	85	0	11	191–377	0.7765	0.7308	0.6897	Complete scoring; no missing/zero genotypes	Highly informative locus with strong contribution to genotype discrimination
gi18465444	83	2	11	122–398	0.5663	0.5033	0.4650	Reproducible scoring with minor missing/zero data	Moderately informative locus contributing to multilocus discrimination
SUGMS43	85	0	4	101–209	0.1412	0.2294	0.2102	Complete scoring; no missing/zero genotypes	Low-to-moderate informativeness; retained for reproducible multilocus profiling
gi18465673	82	3	16	101–356	0.5976	0.7977	0.7686	Reproducible scoring with minor missing/zero data	Most informative locus; major contribution to genotype discrimination

Note. Ho, observed heterozygosity; He, expected heterozygosity; PIC, polymorphism information content. Zeros were treated as missing data. Amplification/scoring quality was assessed from the number of valid genotype calls and missing/zero scores in the curated matrix. The complete list of observed alleles per locus is provided in Supplementary Table S2. The non-retained locus stvia036 was excluded from the final matrix because it did not produce reliable amplification profiles under the tested conditions.

**Table 2:** Distribution of pairwise SSR-derived molecular-affinity categories in the global *Stevia rebaudiana* breeding collection based on the Wang estimator. NR, no detectable positive molecular affinity; IMA, intermediate molecular affinity; HMA, high molecular affinity; CP, pairwise comparisons.

Relatedness Category	Pairwise Comparisons (CP)	Percentage (%)
No detectable positive molecular affinity (NR; *r_xy_* ≤ 0)	1981	55.5
Intermediate molecular affinity (IMA; 0 < *r_xy_* ≤ 0.25)	628	17.6
High molecular affinity (HMA; *r_xy_* > 0.25)	961	26.9
Total	3570	100.0

**Table 3:** Group-level SSR-derived molecular-affinity structure and permutation-based assessment of mean Wang relatedness.

Group	Number of Genotypes	Within-Group Comparisons	NR Comparisons, *n* (%)	IMA Comparisons, *n* (%)	HMA Comparisons, *n* (%)	Empirical *p*-Value	Descriptive Interpretation
Group 1	55	1485	415 (27.9)	290 (19.5)	780 (52.5)	0.002	Compact high-affinity subset. The upper-tailed permutation result supports higher mean Wang relatedness than expected under random reassignment.
Group 2	30	435	341 (78.4)	51 (11.7)	43 (9.9)	0.948	Broader molecular background dominated by NR comparisons. The absence of upper-tailed evidence for excess mean Wang relatedness indicates no detectable enrichment of internal molecular affinity under the tested null model.

NR, no detectable positive molecular affinity; IMA, intermediate molecular affinity; HMA, high molecular affinity. Empirical *p*-values correspond to the permutation-based assessment of mean Wang relatedness under random reassignment of genotypes while preserving group sizes. Molecular-affinity classes are interpreted as operational indicators derived from a reduced five-locus SSR panel and should not be considered definitive genealogical or pedigree assignments.

## Data Availability

The curated SSR genotype matrix, pairwise Wang relatedness matrix, Wang-derived dissimilarity matrix, group assignment file, and R scripts supporting the analyses reported in this study are provided as Supplementary Materials. Additional information related to the broader breeding programme is available from the corresponding author upon reasonable request.

## Supplementary Materials

The following supporting information can be downloaded at: https://www.mdpi.com/article/10.3390/ijms27125277/s1, Table S1: Supplementary files included in the package; Table S2: Summary of retained SSR loci computed from the curated 85-genotype dataset; Table S3: Wang molecular-affinity class summary from the uploaded pairwise file; Table S4: Permutation-based support reported for the Wang relatedness analysis; Table S5: Quality-control summary for consistency between the manuscript and supplementary datasets; Supplementary Figure S1: Representative capillary electropherograms of SSR amplification products in selected *Stevia rebaudiana* genotypes; File S1: R script for reconstructing molecular-affinity class summaries, the Wang relatedness matrix, the Wang-derived dissimilarity matrix, the UPGMA tree and the permutation-based support; IJMS_Supplementary_Materials_Complete.xlsx: Full supplementary workbook; Stevia_85genotypes_5SSR_curated.csv: Curated 85-genotype, five-locus SSR matrix; Wang_pairwise_relatedness_curated.csv: Pairwise Wang relatedness dataset; LynchLi_pairwise_relatedness_curated.csv: Optional comparative pairwise relatedness output based on the LynchLi estimator.

## Abbreviations

The following abbreviations are used in this manuscript:

CP	Pairwise comparisons
cv.	Cultivar
HMA	High molecular affinity
IMA	Intermediate molecular affinity
NR	No detectable positive molecular affinity
SSR	Simple sequence repeat
UPGMA	Unweighted pair-group method with arithmetic mean
*p*	Probability value associated with statistical testing
*r_xy_*	Pairwise relatedness estimated using the Wang estimator
