# Supplementary material for: A Five-Locus SSR Molecular-Affinity Framework Provides Redundancy Context for Previously Identified Elite-Relevant Lines in a ‘Morita II’-Derived Stevia rebaudiana Breeding Collection
Source: Int J Mol Sci. 2026 Jun 10;27(12):5277. doi: 10.3390/ijms27125277 (PMC13299656; doi:10.3390/ijms27125277)
Supplement: Supplementary file 1 [file ijms-27-05277-s001.zip › Supplementary_Materials.pdf]

Supplementary Materials

A Five-Locus SSR Molecular-Affinity Framework Provides Redundancy Context for Previously Identified Elite-Relevant Lines in a Morita II-Derived Stevia rebaudiana Breeding Collection

This supplementary document summarises the supporting datasets generated from the uploaded SSR genotype matrix and pairwise relatedness outputs. The complete editable data tables, matrices and R-ready files are provided in the accompanying Excel workbook and CSV/TXT files.

Table S1. Supplementary files included in the package

File	Content	Purpose
IJMS_Supplementary_Materials_Complete.xlsx	Full workbook with genotype list, SSR matrix, marker statistics, allele frequencies, Wang and LynchLi pairwise relatedness outputs, matrices, summaries, permutation support and QA checklist.	Primary supplementary data file.
Stevia_85genotypes_5SSR_curated.csv	Curated 85-genotype, five-locus SSR matrix with reference group assignment.	R-ready input file.
Wang_pairwise_relatedness_curated.csv	3570 unique pairwise Wang relatedness comparisons with operational molecular-affinity classes and Wang-derived dissimilarity.	Primary pairwise relatedness dataset.
LynchLi_pairwise_relatedness_curated.csv	Optional comparative pairwise relatedness output based on the LynchLi estimator.	Secondary/comparative estimator.
File_S1_R_script_Wang_UPGMA_permutation.R/.txt	R script to reconstruct molecular-affinity class summaries, Wang relatedness matrix, Wang-derived dissimilarity matrix, UPGMA tree and permutation-based support.	Reproducibility file.

Table S2. Summary of retained SSR loci computed from the curated 85-genotype dataset

Locus	Allele column 1	Allele column 2	Valid genotypes (n)	Missing/zero genotypes (n)	Number of alleles	Allele size range (bp)	Observed alleles	Ho	He	PIC	Interpretative note
gi16949765	gi16949765_AL #1	gi16949765_AL #2	85	0	3	99–189	99, 186, 189	0.0353	0.0348	0.0345	Computed from the curated 85-genotype matrix; zeros were treated as missing.
SUGMS28	SUGMS28_AL#1	SUGMS28_AL#2	85	0	11	191–377	191, 194, 200, 203, 206, 209, 212, 215, 239, 245, 377	0.7765	0.7308	0.6897	Computed from the curated 85-genotype matrix; zeros were treated as missing.
gi18465444	gi18465444_AL #1	gi18465444_AL #2	83	2	11	122–398	122, 176, 185, 191, 308, 329, 374, 377, 380, 383, 398	0.5663	0.5033	0.465	Computed from the curated 85-genotype matrix; zeros were treated as missing.
SUGMS43	SUGMS43 AL #1	SUGMS43 AL #2	85	0	4	101–209	101, 201, 205, 209	0.1412	0.2294	0.2102	Computed from the curated 85-genotype matrix; zeros were treated as missing.
gi18465673	gi18465673 AL #1	gi18465673 AL #2	82	3	16	101–356	101, 107, 119, 122, 149, 170, 173, 182, 203, 206, 233, 236, 266, 272, 284, 356	0.5976	0.7977	0.7686	Computed from the curated 85-genotype matrix; zeros were treated as missing.

Table S3. Wang molecular-affinity class summary from the uploaded pairwise file

Estimator	Comparison_context	Comparisons (n)	NR n	NR %	IMA n	IMA %	HMA n	HMA %	Mean relatedness	Median	Minimum	Maximum	SD
Wang	Global	3570	1981	55.49	628	17.59	961	26.92	-0.055	-0.0697	-1.7673	1.0	0.4596
Wang	Within Group 1	1485	415	27.95	290	19.53	780	52.53	0.2295	0.3135	-0.6775	1.0	0.3573
Wang	Between Group 1 and Group 2	1650	1225	74.24	287	17.39	138	8.36	-0.232	-0.1443	-1.7673	1.0	0.3912
Wang	Within Group 2	435	341	78.39	51	11.72	43	9.89	-0.3544	-0.3746	-1.7673	0.8504	0.4819

Table S4. Permutation-based support reported for the Wang relatedness analysis

Analysis_level	Observed/statistical result	Empirical upper-tailed p-value	Interpretation	Source/Note
Global collection	Observed mean Wang relatedness showed upper-tailed departure from random expectation under random reassignment	0.034	Supportive evidence that the observed molecular-affinity pattern was not fully explained by arbitrary group composition	Based on 500 permutations
Within Group 1	Mean Wang relatedness was higher than expected under random reassignment	0.002	Upper-tailed evidence of enriched internal molecular affinity within a compact high-affinity subset	Based on 500 permutations
Within Group 2	Mean Wang relatedness did not show upper-tailed evidence of excess relative to random expectation	0.948	No detectable enrichment of internal molecular affinity under the tested null model; not interpreted as positive evidence of diversification potential	Based on 500 permutations

Table S5. Quality-control summary for consistency between the manuscript and supplementary datasets

QA item	Status	Details
Curated SSR genotypes	Completed	85 genotypes recovered from Stevia_85genotipos_5Loci_final (2)(7).xlsx; Group 1 = 55, Group 2 = 30.
SSR loci	Completed	Five retained loci: gi16949765, SUGMS28, gi18465444, SUGMS43 and gi18465673. Zeros treated as missing for marker summary.
Wang pairwise comparisons	Completed	3570 unique unordered pairwise comparisons recovered from output_wang_Parentesco_Stevia (2)(2).xlsx.
Wang molecular-affinity class counts from pairwise data	Completed	Global: NR = 1981, IMA = 628, HMA = 961; Group 1: NR = 415, IMA = 290, HMA = 780; Group 2: NR = 341, IMA = 51, HMA = 43.
Manuscript count consistency	Completed	Manuscript count consistency: Completed. The manuscript and supplementary files consistently report Group 1 as NR = 415, IMA = 290 and HMA = 780, using the operational thresholds rxy ≤ 0, 0 < rxy ≤ 0.25 and rxy > 0.25.
LynchLi output	Completed	LynchLi pairwise table included as an optional comparative estimator because an uploaded LynchLi output was provided.
Matrix diagonal	Completed	Relatedness matrices use 1.0 on diagonal for display only; summaries and pairwise counts use off-diagonal pairwise comparisons only. Dissimilarity matrix uses 0.0 on diagonal.
R script	Completed	Script reconstructs class summaries, Wang-derived dissimilarity matrix, UPGMA tree and permutation assessment from the curated input files.

Note. The uploaded Wang pairwise file contains 3570 unique off-diagonal pairwise comparisons. Using the operational thresholds r_{xy} <= 0, 0 < r_{xy} <= 0.25 and r_{xy} > 0.25, the computed Group 1 counts are NR = 415, IMA = 290 and HMA = 780.

Relatedness matrix diagonal note. Relatedness matrices in the workbook use 1.0 on the diagonal for display only; all summaries and molecular-affinity class counts were computed from off-diagonal pairwise comparisons. The Wang-derived dissimilarity matrix uses 0.0 on the diagonal.
