# Supplementary material for: A Five-Locus SSR Molecular-Affinity Framework Provides Redundancy Context for Previously Identified Elite-Relevant Lines in a ‘Morita II’-Derived Stevia rebaudiana Breeding Collection
Source: Int J Mol Sci. 2026 Jun 10;27(12):5277. doi: 10.3390/ijms27125277 (PMC13299656; doi:10.3390/ijms27125277)
Supplement: Supplementary file 1 [file ijms-27-05277-s001.zip › Supplementary_Figure_S1.pdf]

Supplementary Figure S1. Representative capillary electropherograms of SSR amplification products in selected *Stevia rebaudiana* genotypes

Representative electropherograms obtained after capillary electrophoresis using a SeqStudio Genetic Analyzer and allele scoring in GeneMarker v3.0. The profiles illustrate SSR fragment detection and allele-size calling for selected loci included in the molecular-affinity analysis. These profiles are shown as representative examples of fragment detection and should not be interpreted as agarose gel or PAGE profiles.

Methodological context for Supplementary Figure S1

Item	Description
**Sample set**	The source report records 87 Stevia leaf samples identified by line code and processed for SSR analysis.
**PCR optimisation**	Six SSR loci were evaluated; stvia036 was not retained because it failed under the tested amplification conditions. The final molecular-affinity analysis used five retained SSR loci.
**PCR product check**	Agarose gel electrophoresis was used as an initial amplification/optimisation check for PCR products; this was not the allele-scoring platform.
**Final fragment detection**	Allele-size profiles for retained SSR loci were visualised using the SeqStudio genetic analyser and scored from capillary fragment-analysis outputs.
**Purpose of this figure**	The panels document representative capillary electropherogram profiles and allele-size calls used to support SSR fragment detection in the revised supplementary material.

Panel summary

Panel	SSR locus	Representative allele-size information shown in the electropherogram
A	SUGMS28	Representative allele calls around the 199-215 bp range.
B	SUGMS43	Representative allele calls around the 200-206 bp range.
C	gi18465444	Representative allele calls around the 376-397 bp range.
D	gi16949765	Representative allele calls around the 185-189 bp range.
E	gi18465673	Representative allele calls around the 234-266 bp range.

Note. Panel labels and allele-size calls are retained from the original electropherogram screenshots. The images are intended as representative visual evidence of fragment detection and allele-size calling, not as a complete presentation of all genotyped samples.

Panel A. SUGMS28

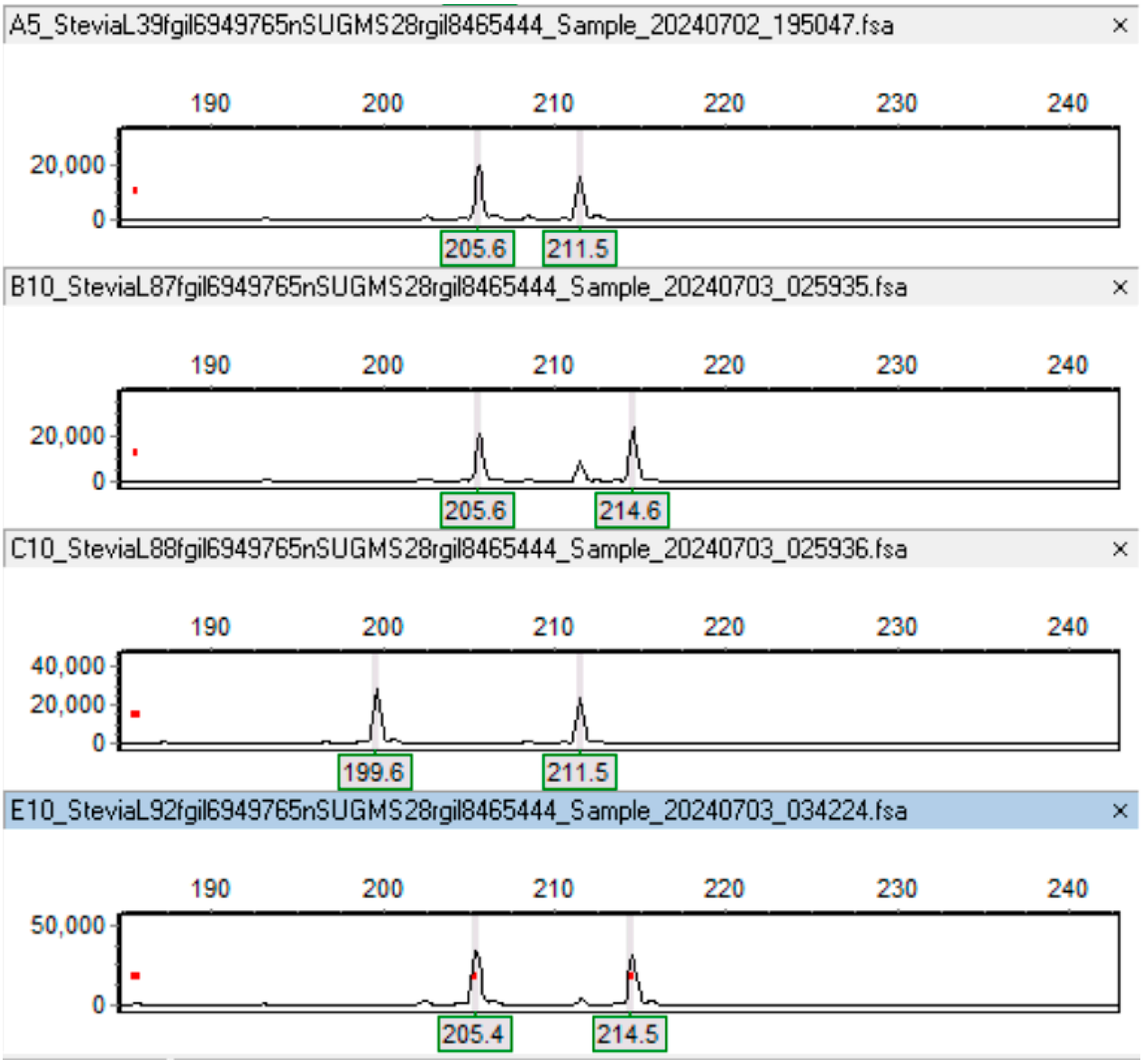

Panel A shows representative capillary electropherogram profiles for SUGMS28. Green allele-size boxes indicate called fragment sizes used for SSR genotype scoring. Representative allele calls around the 199-215 bp range.

Panel B. SUGMS43

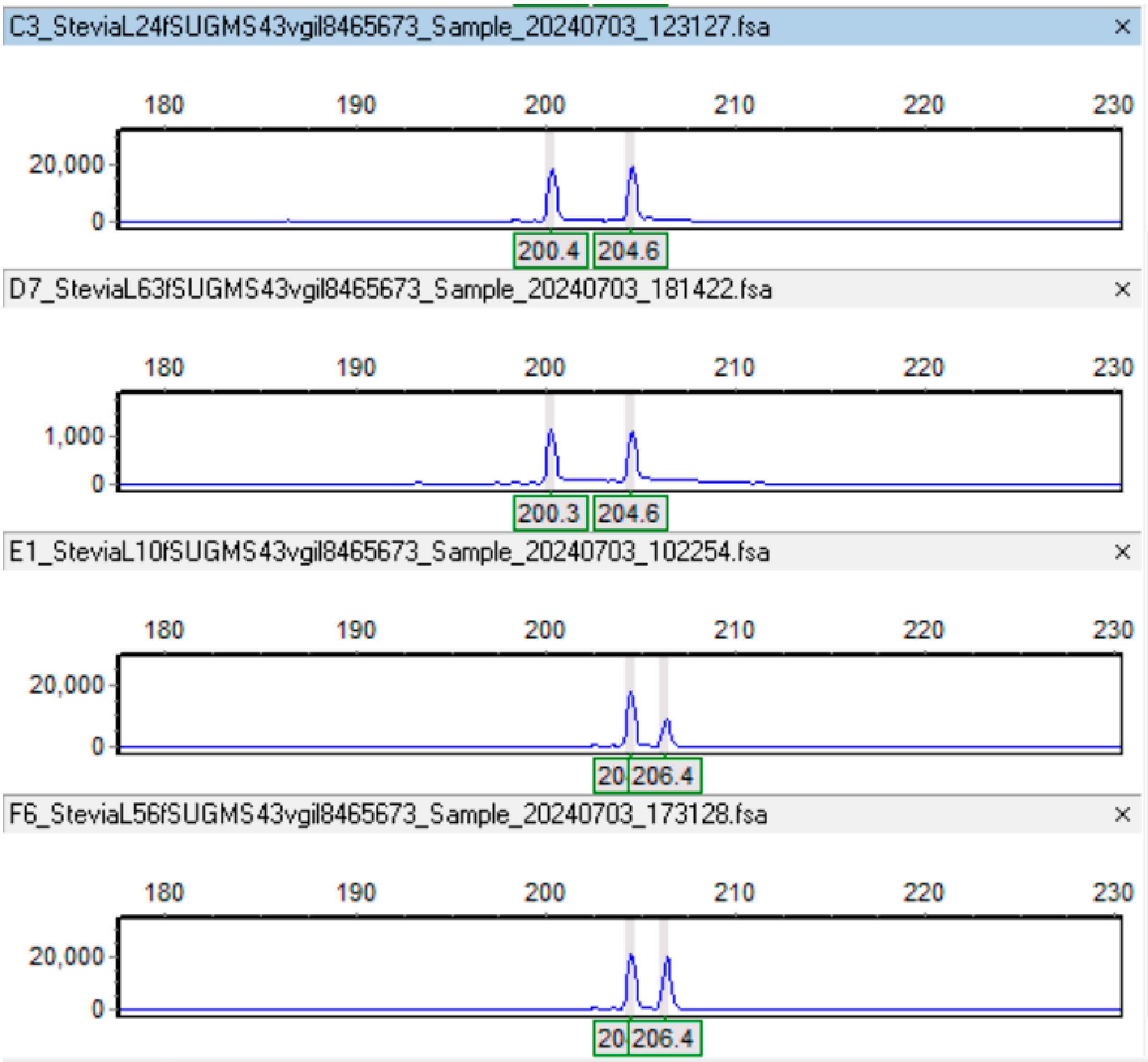

Panel B shows representative capillary electropherogram profiles for SUGMS43. Green allele-size boxes indicate called fragment sizes used for SSR genotype scoring. Representative allele calls around the 200-206 bp range.

Panel C. gi18465444

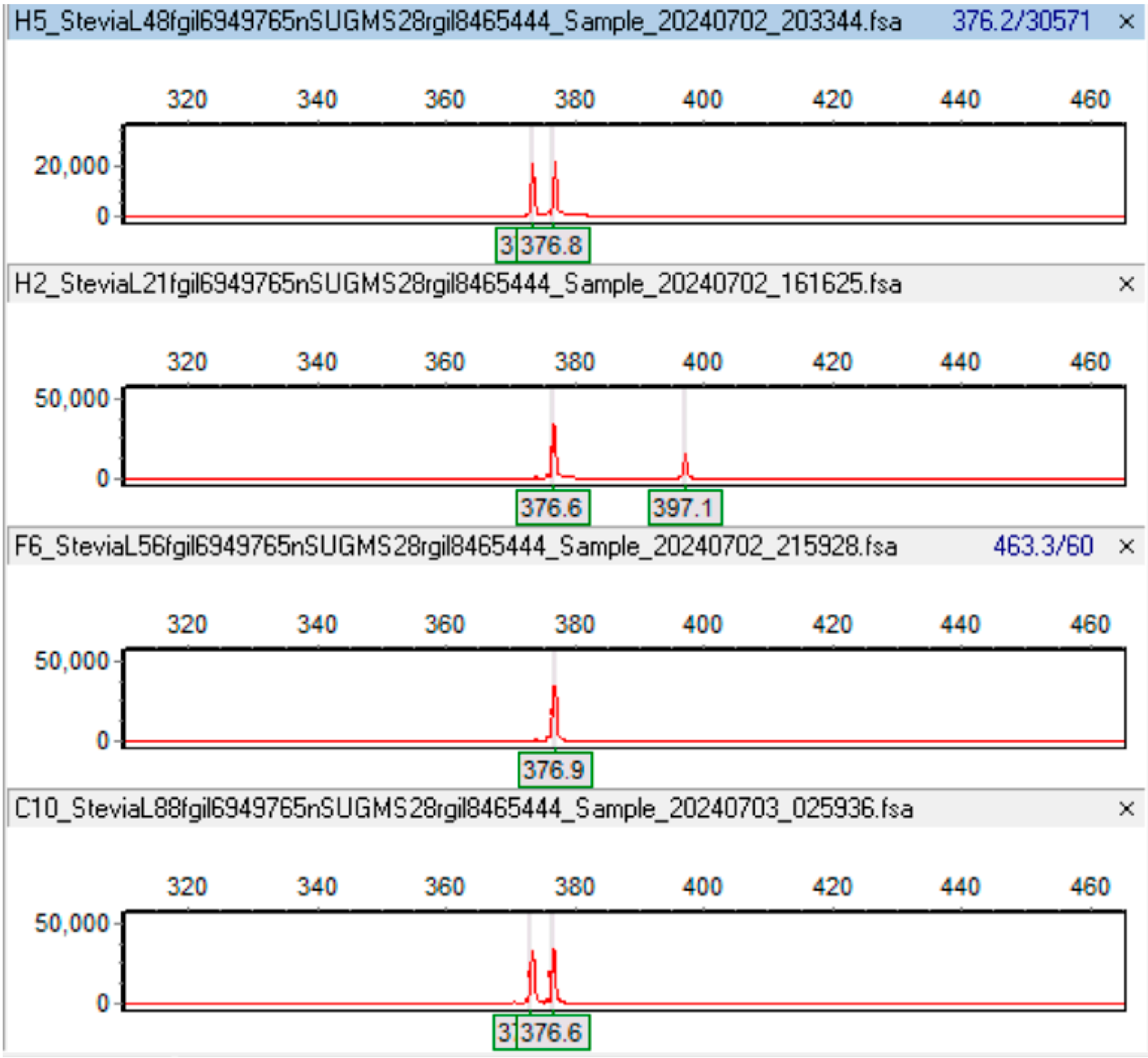

Panel C shows representative capillary electropherogram profiles for gi18465444. Green allele-size boxes indicate called fragment sizes used for SSR genotype scoring. Representative allele calls around the 376-397 bp range.

Panel D. gi16949765

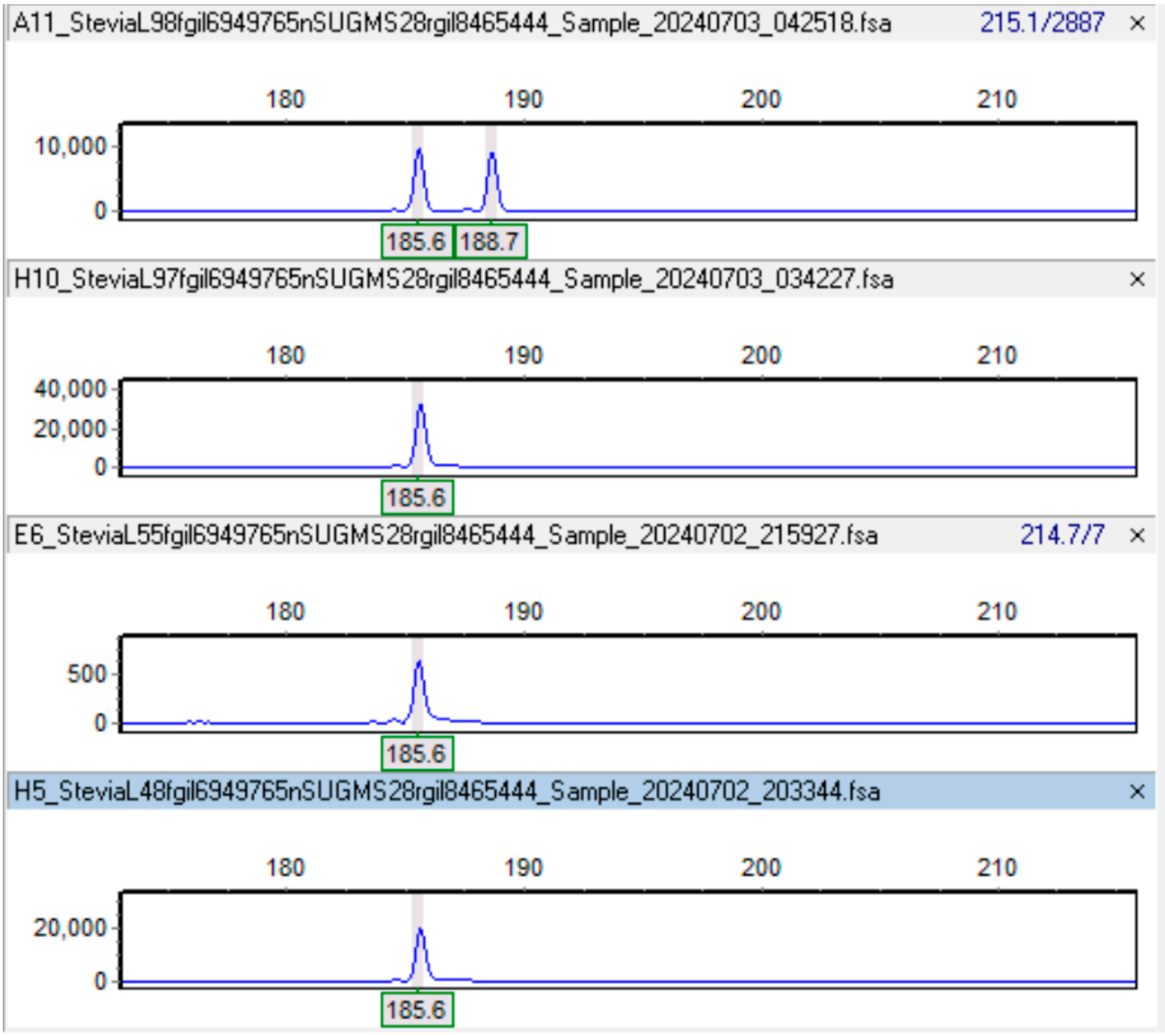

Panel D shows representative capillary electropherogram profiles for gi16949765. Green allele-size boxes indicate called fragment sizes used for SSR genotype scoring. Representative allele calls around the 185-189 bp range.

Panel E. gi18465673

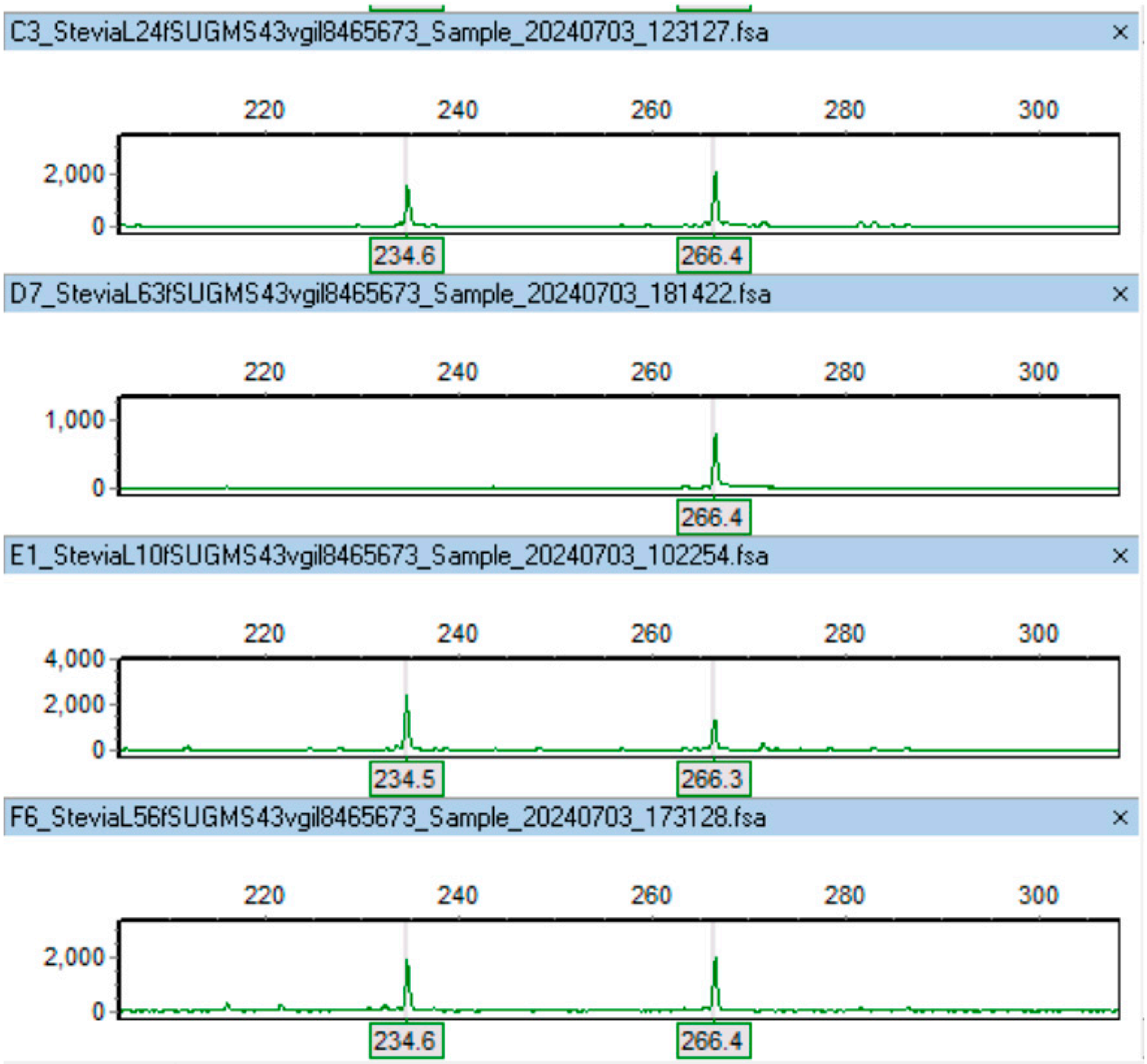

Panel E shows representative capillary electropherogram profiles for gi18465673. Green allele-size boxes indicate called fragment sizes used for SSR genotype scoring. Representative allele calls around the 234-266 bp range.

Interpretive note. This Supplementary Figure S1 focuses on capillary electropherogram outputs because these profiles correspond to fragment sizing and allele calling for the SSR molecular-affinity dataset.
